# Affective cognition in eating disorders: a systematic review and meta-analysis of the performance on the “Reading the Mind in the Eyes” Test

**DOI:** 10.1007/s40519-022-01393-8

**Published:** 2022-04-06

**Authors:** Antonio Preti, Sara Siddi, Enrica Marzola, Giovanni Abbate Daga

**Affiliations:** 1 Eating Disorders Unit, Department of Neuroscience “Rita Levi Montalcini”, University of Turin, via Cherasco 15, 10126 Turin, Italy; 2 Parc Sanitari Sant Joan de Déu, Fundació Sant Joan de Déu, Institut de Recerca Sant Joan de Déu, Sant Boi de Llobregat, Universitat de Barcelona, Dr. Antoni Pujadas, 42, 08830 Sant Boi de Llobregat, Barcelona, Spain

**Keywords:** Social cognition, Theory of Mind, Anorexia nervosa, Bulimia nervosa, Binge eating disorder, Meta-analysis

## Abstract

**Background:**

The Reading the Mind in the Eyes Test (RMET) is listed in the National Institute of Mental Health’s Research Domain Criteria as a tool apt to measure the understanding of others’ mental states. People diagnosed with anorexia nervosa (AN) showed poorer performances on the RMET than healthy controls. Less data are available concerning other eating disorders.

**Methods:**

Systematic review of four major databases from inception to July 15, 2021 following the PRISMA guidelines. Meta-analysis of cross-sectional observational studies comparing the scores of the RMET between patients with eating disorders and age- and-gender matched control groups.

**Results:**

Out of 21 studies, we retrieved 29 independent samples of patients diagnosed with an eating disorder. Patients with active AN (*n* = 580) showed worse performances on the RMET than controls (*n* = 1019). Year of publication accounted for 61% of the (substantial: *I*^*2*^ = 81%) heterogeneity in the meta-analysis. Earlier studies were more likely to find worse performances on the RMET of patients with active AN than later studies. Patients with bulimia nervosa (*n* = 185) performed poorly as compared to controls (*n* = 249), but the results were not statistically significant on the random-effect model. Obese patients with binge-eating disorder (*n* = 54) did not differ on the RMET from obese controls (*n* = 52). Patients with eating disorder not otherwise specified (*n* = 57) showed minimal differences compared to controls (*n* = 96). Study quality was good in six studies only.

**Conclusions:**

Patients with eating disorders do not suffer from an impaired understanding of others’ mental states, except for a still-to-be-identified subgroup of patients with active AN.

**Level of evidence:**

I, systematic review and meta-analysis.

**Supplementary Information:**

The online version contains supplementary material available at 10.1007/s40519-022-01393-8.

## Introduction

Current neuroscience posits that there are brain systems dedicated to the perception and interpretation of others’ actions [1, 2]. Within this framework, the US National Institute of Mental Health’s (NIMH) Research Domain Criteria (RdoC) have identified the understanding of mental states as a core domain in the perception and understanding of others. This domain, akin to the concept of “Theory of Mind” (ToM; [3]), covers all the processes that are involved in the judgment and/or attribution of mental states to other animated entities so as to predict or interpret their behaviors [4].

The Reading the Mind in the Eyes Test (hereinafter the RMET) is listed in the NIMH RdoC as a tool apt to measure the understanding of others’ mental states. In its final format, the RMET consists of 36 pictures of the eyes region on the face drawn from magazines and newspapers [5]. The candidate is presented with the pictures alongside four words that are supposed to describe what the pictured person is thinking or feeling. One word is the expected correct designator and the other three words are foils. The candidate can consult a glossary to get a better understanding of the meaning of the words accompanying the picture. The score is the sum of all correct answers. The RMET is thought to measure a single factor [6, 7], although some authors have found a multidimensional factor structure of the RMET [8]. Performance on the RMET depends on intelligence quotient (IQ), and in particular, verbal IQ [9], and its psychometric properties vary according to the samples and version, especially its reliability and its links with self-report measures of empathy [10]. Women outperform men on the RMET [10, 11], and there is some evidence that accuracy may vary between same-race targets and other-race targets [12]. Activation in some cortical areas and related structures were seen during the execution of the RMET, in particular, the superior temporal sulcus, inferior frontal gyrus, medial prefrontal cortex, hippocampus, and cerebellum [13, 14].

The RMET was devised to assess social cognition in autistic people [5], and in fact, the task appears best suited for testing populations at the lower end of performances on the measured ability [7]. Nevertheless, over time, it has become a popular task for the assessment of emotion recognition, understanding of complex mental states, and ToM capacity in both clinical and non-clinical populations (e.g., [15, 16]). The RMET assesses the ability to recognize complex mental states as expressed by human eyes. However, most ToM tasks measure the ability of the candidate to infer non-emotional mental states, such as intentions or beliefs, from contextual information or dynamic behavioral cues. Thus, some authors questioned the specificity of the RMET as ToM task, suggesting it is principally a measure of emotion recognition [17]. Nevertheless, general consensus distinguishes between a cognitive component of ToM, which is the ability to attribute mental states to others via perspective taking, and an affective component of ToM, which is based on the ability to infer another person’s emotions via facial or body expressions [18, 19]. The RMET is a reasonably apt measure of the affective component of ToM, since emotion recognition and appraisal contribute to the attribution of mental states to others. Moreover, test performances on the RMET were consistently related to the activation of the dorsomedial prefrontal cortex and the temporoparietal junction [20], which are typically related to ToM tasks [19]. Test performances on the RMET were as well related to the within-network connectivity of the right posterior superior temporal sulcus [21], and the gray matter density in the left posterior superior temporal sulcus and its functional connection with the amygdala [22], which are implied in social perception, another component of ToM [19].

Superior performances of controls over autistic people are the most replicated finding with the RMET [23]. People diagnosed with schizophrenia also show poorer performances on the RMET than healthy controls [24], with effect sizes comparable to those observed in autistic people [25]. Conflicting results were found in samples of patients diagnosed with borderline personality disorder, with some studies finding enhanced performances compared to controls [26], and other studies finding the reverse [27–29]. Even less consistent findings were reported in patients with bipolar disorder [30–33], in those with major depressive disorder [34, 35], and in patients with obsessive–compulsive disorder [36, 37].

The impairment in the abilities involved in the RMET was related to deficits in social functioning and poor insight in schizophrenia [38, 39]. Impaired social functioning is also a core characteristic of autism spectrum disorder [40]. Individuals with anorexia nervosa (AN), too, are known to suffer from a deficit in social functioning [41–44]. They also experience a poor insight about their symptoms, sometimes so severe to reach the level of delusion [45]. Moreover, people diagnosed with AN manifest neuropsychological features similar to those that are observed in autism spectrum disorder, such as weak central coherence, cognitive inflexibility, and problem in emotion recognition [46]. Unsurprisingly, people diagnosed with AN showed poorer performances on the RMET than healthy controls, with larger effect sizes in acute patients as compared to recovered ones [47]. Social anxiety, poor social support, and interpersonal difficulties were reported also in patients with bulimia nervosa (BN; [41, 48]), who also showed reduced performances on the RMET compared to controls [47]. People with binge-eating disorder (BED) or with eating disorder not otherwise specified (EDNOS) were less often investigated on their performances on the RMET [49, 50], although both people with BED [51], and EDNOS [52], experience interpersonal difficulties. Moreover, BED and EDNOS displaying disordered eating with binge eating may evolve into obesity [53]. There is some evidence that people with obesity exhibit some degree of impairment on instruments assessing emotion recognition and ToM [54]. Thus, the investigation of affective cognition in patients diagnosed with BED and EDNOS is justified.

Conflicting results emerged in recent studies regarding the capacity to understand mental states in people diagnosed with an eating disorder. Some studies reported that patients with AN were as good as controls in responding to the RMET [55, 56], and similar findings were reported for patients diagnosed with BN [57, 58]. It is possible that the bias towards positive findings, i.e., the observation of a difference with controls—the most frequent cause of publication bias—, led to a focus on deficient performances on the RMET of people with eating disorders in earlier studies. For this reason, the role of the year of publication should be taken into account when investigating the performances on the RMET of patients with eating disorders. Another element inextricably interlaced with the year of publication is the role played by the criteria for diagnosis in the enrollment of samples. Following its introduction, it became evident that the samples of patients diagnosed with AN or BN according to the fifth edition of the Diagnostic and Statistical Manual of Mental Disorders (DSM-5) may include less severe cases than those included in samples that were diagnosed according to the more restrictive criteria of the DSM-IV. Indeed, cases that would have been classified under the label of EDNOS according to the DSM-IV are now diagnosed under one major category (AN, BN, BED) according to the DSM-5 [59]. Less severe cases might be less impaired on affective cognition, thus leading to fewer differences in the RMET between cases and controls in studies done according to DSM-5 criteria than in previous studies based on past DSM criteria.

Social cognition takes on an important role in behavioral disorders and may become the target of therapeutic and rehabilitative interventions [60, 61]. Thus, a reappraisal of the performances of people with eating disorders on a task aimed at measuring a core component of social cognition—the understanding of others’ mental states—is worthwhile.

So far, the performances on the RMET of patients diagnosed with an eating disorder were summarized in a meta-analysis focusing on those with AN and BN, which dates back to 2016 and mostly included studies done before the DSM-5 entered into standard use in research [47]. The topic was also briefly covered in a systematic review devoted to mentalization in patients with eating disorders [62], and in a meta-analysis investigating autistic features in patients with anorexia nervosa [46]. The demonstration that a deficit of affective cognition is specific to AN and is less evident or absent in other eating disorders would support a role for it in the etiology of AN. Nevertheless, it cannot be excluded that a deficit of affective cognition in AN is merely a reflection of malnutrition. The impact of body mass index (BMI), as a proxy for the severity of AN, on the performance on the RMET might serve the purpose of investigating the role of malnutrition in AN. Differences in the performances on the RMET between samples of recovered AN and those with active AN would further corroborate a role for malnutrition.

### Aims

This systematic review and meta-analysis was set out to: (a) summarize the available evidence on the performances on the RMET of people diagnosed with an eating disorder; (b) evaluate the quality of available evidence; (c) highlight current strengths and evidence gaps. We focused on studies that included either community or clinical samples irrespectively of gender or age; with performances on the RMET as an outcome; that had eating disorders as the criterion of exposure; that reported information by comparison with controls without eating disorders.

## Methods

This meta-analysis was done according to the indications of the Preferred Reporting Items for Systematic Reviews and Meta-Analyses (PRISMA) guidelines [63, 64]. We searched PubMed/Medline, the Cochrane library, the Excerpta Medica Database (EMBASE), and PsycInfo without time limitations from inception until 15 July 2021. A combination of the following key terms was used: Reading the Mind in the Eyes Test, Reading the Mind in the Eyes, RMET, AND eating disorders, anorexia nervosa, bulimia nervosa, binge-eating disorder, eating disorder not otherwise specified, other specified feeding or eating disorder.

Studies were included when they were published in a peer-reviewed journal; abstracts and unpublished theses were excluded. Indeed, there is evidence that selection bias is higher in unpublished literature than in published literature [65, 66]. Based on the knowledge of the languages of the evaluators, articles that were written in English, Spanish, French, or Italian were assessed. However, there is some evidence that a search of English language literature is enough to produce results that are similar to those that can be retrieved, with more time and effort, from reviews based on comprehensive searches free of language restrictions [65].

**Fig. 1:**
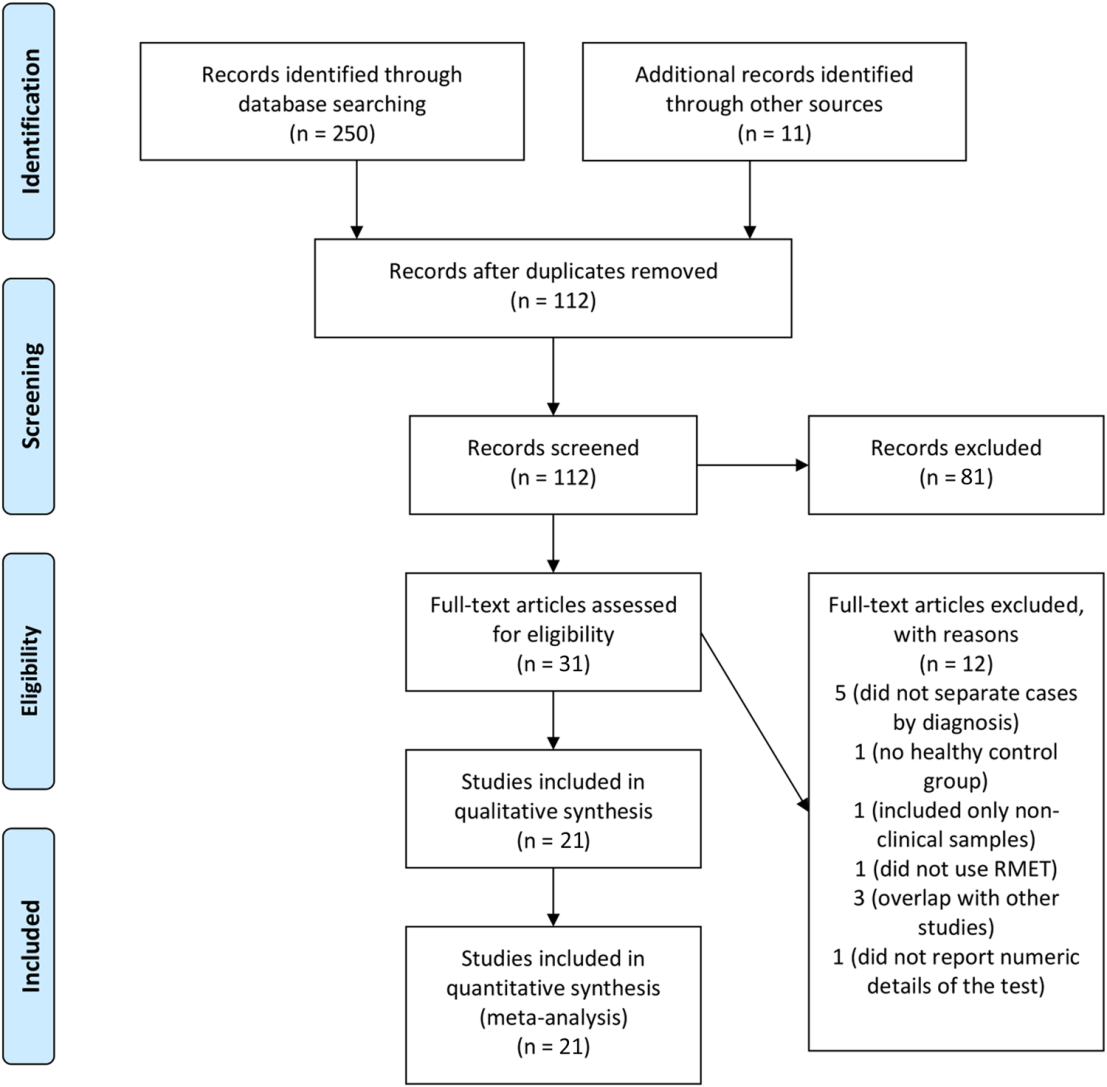
PRISMA Flow chart

Two evaluators performed the search. The evaluators had 15 and 30 years of experience, respectively, in conducting research in the behavioral field, and had 6 and 10 years of experience in conducting systematic reviews and meta-analysis. The list of the retrieved articles was inspected by the two evaluators to establish whether the articles were congruent with the search criteria. Duplicates were eliminated. Discrepancies were solved by discussion. Collected articles were then thoroughly re-examined for content and their references section was scanned to identify missed studies. The same procedure was applied to the scanning of the additional sources (systematic reviews/meta-analyses). When a group published more than one report with a probable overlap of the samples, the study with the largest sample was included (Fig. 1).

Two evaluators extracted the following data from each article: author(s) and year of publication; the geographical location of the study; condition under test; criteria for diagnosis; sample size and composition by gender and age; body mass index; duration of illness; mean scores and standard deviation (SD) of the participants on the RMET by group. When the authors reported the median and the interquartile range, the median was considered equivalent to the mean and the interquartile range was converted to SD by dividing it by 1.35 (as per Sect. 6.5.2.5 “Interquartile ranges” of the Cochrane handbook; [67]). Discrepancies in extraction of data were solved by discussion.

The main characteristics of the studies are summarized in Table 1; in “Results”, studies are grouped by diagnosis.

**Table 1:** General characteristics of the studies

Study	Year	Location	Condition	Criteria for diagnosis	Sample	BMI	Duration of illness (years)
Harrison et al. [81]	2009	London, UK	Anorexia nervosa	DSM-IV	AN: 20 Women: 20 Age: 26.25 (5.73) HC: 20 Women: 20 Age: 28.35 (8.46)	AN: 15.81 (1.15) HC: 21.78 (1.61)	AN: 7.2 (3.2)
Russell et al. [82]	2009	London, UK	Anorexia nervosa	DSM-IV	AN: 22 Women: 22 Age: 26.7 (4.8) HC: 22 Women: 22 Age: 30.3 (6.5)	AN: 15.26 (1.2) HC: 26.2 (2.0)	9.5 (5.0)
Harrison et al. [83]	2010	London, UK	Anorexia nervosa	DSM-IV	Acute AN: 50 Women: 50 Age: 26.7 (9.82) Recovered AN: 35 Women: 35 Age: 29.0 (10.62) HC: 90 Women: 90 (?) Age: 28.5 (9.93)	Acute AN: 15.38 (1.83) Recovered AN: 21.15 (1.76) HC: 21.61 (1.89)	Acute AN: 9.23 (9.27) Recovered AN: 5.47 (2.90)
Oldershaw et al. [84]	2010	London, UK	Anorexia nervosa	DSM-IV	AN: 40 Women: 37 Age: 27.3 (10.0) Recovered AN: 27 Women: 23 Age: 29.9 (7.7) HC: 47 Women: 37 Age: 29.8 (8.0)	AN: 16.6 (1.3) Recovered AN: 20.8 (2.0) HC: 23.0 (2.8)	AN: 7.4 (8.5) Recovered AN: 5.6 3.8
Adenzato et al. [85]	2012	Brescia, Italy	Anorexia nervosa	DSM-IV	AN: 30 (AN-R: 16, AN-BP: 14) Women: 30 Age: 19.7 (6) HC: 32 Women: 32 Age: 20.5 (3)	AN: 15.0 (1.7) HC: 20.2 (1.4)	AN-R: 1.7 AN-BP: 5.8
Kenyon et al. [57]	2012	London, UK	Bulimia nervosa	DSM-IV TR	BN: 48 Women: 48 Age: 28.0 (7.7) EDNOS BN: 34 Women: 33 Age: 27.6 (6.9) HC: 57 Women: ? Age: 24.0 (2.6)	BN: 24.5 (7.1) EDNOS BN: 25.0 (6.3) HC: 22.7 (3.2)	BN: 10.7 (8.1) EDNOS BN: 9.9 (6.5)
Medina-Pradas et al. [50]	2012	Barcelona, Spain	Anorexia nervosa Bulimia nervosa EDNOS	DSM-IV TR	AN: 44 Women: 44 Age: 26.80 (5.70) BN: 30 Women: 30 Age: 26.80 (6.10) EDNOS: 23 Women: 23 Age: 26.02 (8.20) HC: 39 Women: 39 Age: 26.04 (14.70)	AN: 15.80 (1.70) BN: 20.90 (2.60) EDNOS: 25.80 (8.30) HC: 21.30 (2.10)	AN: 9.90 (6.60) BN: 10.30 (5.20) EDNOS: 7.30 (6.40)
Tapajóz Pereira de Sampaio et al. [86]	2013	Buenos Aires, Argentina	Anorexia nervosa Bulimia nervosa	DSM-IV	AN: 24 Women: 24 Age: 24.5 (7.6) BN: 24 Women: 24 Age: 24.4 (6.0) HC: 24 Women: 24 Age: 25.2 (6.9)	AN: 18.1 (1.8) BN: 25.0 (6.5) HC: 21.5 (1.8)	AN: 7.8 (5.9) BN: 7.7 (6.3)
Laghi et al. [87]	2015	Rome, Italy	Anorexia nervosa	DSM-IV TR	AN-R: 40 Women: 40 Age: 14.93 (1.48) HC: 40 Women: 40 Age: 14.88 (0.56)	AN-R: 15.76 (1.45) HC: 21.85 (2.63)	AN-R: 1.28 (1.04)
Jermakow and Brzezicka [88]	2016	Warsaw, Poland	Anorexia nervosa	ICD-10	AN: 11 Women: 11 Age: 26.8 (4.3) HC: 33 Women: 33 Age: 21.3 (1.4)	AN: 14 to 20 HC: unavailable	Unavailable
Kucharska et al. [55]	2016	Cardiff, UK	Anorexia nervosa	ICD-10 DSM-IV TR	AN: 25 Women: 25 Age: 27.1 (6.3) HC: 25 Women: 25 Age: 24.5 (5.2)	AN: 17.6 (2.2) HC: 23.4 (3.6)	AN: 9.7 (6.6)
Aloi et al. [49]	2017	Catanzaro, Italy	Binge eating disorder	DSM-5	Obese BED: 22 Women: 18 Age: 44 (11) Obese not BED: 20 Women: 9 Age: 50 (8)	BED: 36.9 Not BED: 38.2	Unavailable
Bentz et al. [89]	2017	Copenhagen, Denmark	Anorexia nervosa	ICD-10	First episode AN: 43 Women: 43 Age: 16.1 (1.5) Recovered AN: 28 Women: 28 Age: 18.4 (1.6) HC: 41 Women: 41 (?) Age: 17.7 (2.2)	First episode AN: 16.6 (1.2) Recovered AN: 21.3 (1.8) HC: 22.0 (2.6)	Unavailable
Leppanen et al. [90]	2017	London, UK	Anorexia nervosa	DSM-5	AN: 30 Women: 30 Age (median): 24.5 HC: 29 Women: 29 Age (median): 25.0	AN (median): 16.13 HC (median): 22.21	Unavailable
Redondo and Herrero-Fernández [91]	2018	Leioa, Spain	Anorexia nervosa	DSM-IV TR	AN: 38 Women: 38 Age: 21.9 (5.30) HC: 321 Women: 321 Age: 20.0 (2.05) ?	Unavailable	Unavailable
Nalbant et al. [56]	2019	Ankara, Turkey	Anorexia nervosa	DSM-5	AN: 32 Women: 32 Age: 15.2 (1.6) HC: 32 Women: 32 Age: 15.2 (1.7)	AN: 16.6 (1.5) HC: unreported	AN: 1 (0.1)
Rothschild-Yakar et al. [92]	2019	Tel Hashomer, Israel	Anorexia nervosa	DSM-5	AN: 41 (AN-R: 29 AN-BP: 12) Women: 41 Age: 17.58 (2.57) HC: 53 Women: 53 Age: 17.63 (2.39)	AN: 18.04 (1.72) HC: 21.21 (2.46)	Unavailable
Sacchetti et al. [93]	2019	London, UK	Bulimia nervosa	DSM-5	BN: 53 Women: 50 Age: 30.60 (8.91) HC: 87 Women: 76 Age: 29.14 (8.65)	BN: 23.55 (6.09) HC: 21.63 (2.72)	Unavailable
Turan et al. [94]	2019	Izmir, Turkey	Binge eating disorder	DSM-5	Obese BED: 32 Women: 18 Age: 15 (1.43) Obese not BED: 32 Women: 17 Age: 14.81 (1.65)	BED: 34.58 Not BED: 32.72	Unavailable
Konstantakopoulos et al. [58]	2020	Athens, Greece	Anorexia nervosa Bulimia nervosa	DSM-IV TR	AN: 46 Women: 46 (AN-R: 30 AN-BP: 16) Age (median): 26.0 BN: 30 Women: 30 Age (median): 24.0 HC: 42 Women: 42 Age (median): 26.0	AN (median): 16 BN (median): 19.5 HC (median): 20.7	AN (median): 5 BN (median): 4
Cortés-García et al. [95]	2021	[Unknown], [unknown]	AN	DSM-IV	AN: 44 Women: 44 Age: 15.18 (1.46) HC: 129 Women: 129 Age: 15.32 (1.17)	Unavailable	Unavailable

*AN* anorexia nervosa, *AN-R* AN restrictive, *AN-BP* AN binge-purge, *BN* bulimia nervosa, *BED* binge-eating disorder, *EDNOS* eating disorders not otherwise specified, *HC* healthy controls

The risk of bias was assessed using a validated checklist published by the US National Heart, Lung and Blood Institute for case–control studies [URL: www.nhlbi.nih.gov/health-topics/study-quality-assessment-tools, accessed on 20 July 2021].

The procedure was implemented according to an internal protocol.

### Statistical analysis

The effect size of the differences between cases and controls was calculated as bias-corrected standardized mean (i.e., Hedges's g and 95% confidence interval) and computed so that a negative value indicated an unfavorable outcome (e.g., defective performance on the RMET) [68]. According to a largely agreed rule of thumb, values of 0.20, 0.50, and 0.80 were assumed to be the thresholds for small, medium, and large effect sizes [69].

The within-study variance was estimated with the empirical Bayes estimator [70], and its 95% confidence interval (CI) was calculated using the Q-Profile method [71], with Hartung and Knapp correction for random-effects models [72]. The Cochran’s Q and I^2^ statistics were used to estimate heterogeneity [73]. Significant Q statistics (i.e., *p* < 0.05) was interpreted as suggestive of heterogeneity. *I*^*2*^ values between 40 and 60% were considered suggestive of moderate heterogeneity, while values above 75% were considered indicative of substantial heterogeneity [74]. The radial plot was used to assess model adequacy [75], and effect size sampling variance. For each study, the observation of a large standardized residual (above 2, as a rule of thumb) suggests that the study does not fit the assumed model (i.e., it may be an outlier). Publication bias was investigated using the funnel plot and related statistics when there were 10 or more studies [76].

We reported both the fixed-effects and the random-effects models in both tabular and graphical (forest plot) forms. Fixed-effects models estimate a common effect, which is valid only for the studies included in the meta-analysis [77]. The random-effects models aim to provide inference about the average effect in the entire population from which the studies are expected to be drawn. Essentially, the fixed-effects model assumes that variance depends principally on sampling error, while the random-effects model takes into account heterogeneity of the studies, i.e., the fact that the effects that are estimated from the studies come from a distribution of true effects which depend on a source of variability that is not limited to sampling error [78]. In the case of 10 or more studies, we also reported an approximate prediction interval from the random-effects model for a new study, which was calculated according to Higgins et al. [79]. Prediction interval is helpful to establish whether the results of the meta-analysis will hold in future studies.

Sensitivity analysis included the application of the trim-and-fill method to ascertain the impact of publication bias on the premise that the most extreme results are not published. The trim-and-fill method recalculates the effect size by the imputation of the missing studies to produce a symmetrical funnel plot [80]. When there were enough studies (*n* ≥ 10), we also recalculated the effect size by excluding outliers that were eventually revealed by the radial plot. We also used meta-regression to evaluate the impact of the following variables: year of publication; criteria for diagnosis; sample size; gender; age; body mass index; duration of illness; quality of the studies.

Meta-analysis was carried out with R (version 4.0.2) using the following packages: ‘metafor’ (version 2.4-0) and ‘meta’ (version 4.14-0).

## Results

The initial search across the four bibliographic databases yielded 250 records. Additional 11 references were retrieved from past publications on the topic already known to the authors. After the exclusion of duplicates recurring in the databases, 112 articles were screened, and 31publications were inspected after excluding the articles (*n* =  81) that were judged unrelated to the topic on the basis of their title and abstract (see Fig. 1).

Twelve articles were excluded because either they had overlapping samples, or included non-clinical samples only, did not separate cases by diagnosis, did not use the RMET, or did not report numeric details (see the list of excluded studies in the supplemental material).

Overall, we retrieved 21 studies [49, 50, 53–56, 83–95]. These 21 studies included 29 independent samples of patients diagnosed with an eating disorder: 20 samples included people diagnosed with AN (17 with acute cases, and 3 with recovered cases); 5 were samples of patients diagnosed with BN; there were 2 samples of patients diagnosed with EDNOS (one specifically with EDNOS BN) and 2 samples of patients diagnosed with BED. These samples were compared with 20 independent samples of gender- and age-matched healthy controls.

Compared to a past meta-analysis on the topic [47], this systematic review and meta-analysis includes more than twice the number of studies and the sample size. Another systematic review [62], which also reported data on the RMET in eating disorders, retrieved 14 studies against 21 in the present review. A recent meta-analysis investigating autistic features in patients with anorexia nervosa and reporting data on the RMET [46], retrieved 10 independent samples only as against 17 samples in this meta-analysis. All studies included in the most recent past systematic reviews and meta-analyses on the topic are included in the present review, which also offers substantial additional information.

Table 1 lists the main characteristics of the included studies. Among these, 8 were from the UK, 3 from Italy, 2 from Spain and 2 from Turkey, and 1 each from Argentina, Denmark, Greece, Israel, and Poland. We were unable to determine the location of one study [95]. We were unable to retrieve studies from Asia, Africa, or Oceania. Thus, there is an ethnic bias in the studies about the performances on the RMET of people with eating disorders.

Sample size varied from 11 to 53 in the clinical groups (on average, 33 ± 10), and from 20 to 129 in the control groups (45 ± 25), after the exclusion of an outlier (*n* = 321 in [91]), which was, nevertheless, included in the meta-analysis.

The samples included quite exclusively girls or women (96% of all included participants).

Age varied widely in the included samples, from 15 to 44 years old in the clinical samples and from 15 to 50 years old in the control samples. Overall, there were six samples of underaged patients (< 18 years old) and just one sample of clinical participants aged > 30 years old. We used body mass index (BMI) as a proxy of the severity of the disorder in patients with AN. As expected, BMI was lower in patients diagnosed with AN (17.2 ± 2) than in patients with BN (22.7 ± 2.4; t = − 5.07; df = 20; *p* < 0.0001), and it was lower in patients with active AN (16.3 ± 1) than in those with recovered AN (21.1 ± 0.2; *t* = 8.06; df = 15; *p* < 0.0001). Patients with EDNOS were in the same range as those with BN (25.4 ± 0.5; *t* = 1.51; df = 5; *p* = 0.19), and those with BED were obese (35.7 ± 1.6). Duration of illness was reported for 20 samples and varied from 1 to 10 years.

Study quality was judged poor in 8 studies, fair in 7 studies, and good in 6 studies. Lack of sample size justification and the lack of blinding of the assessors were the most often observed shortcomings of the studies (Table A in the supplementary material).

Poor studies were equally distributed across time, while good studies were more often observed in recent years, from 2016 on.

### Results of the meta-analysis

As a preliminary analysis, we estimated the raw, untransformed mean performance of the contrast group (putatively healthy controls) in the AN and BN studies. Three studies reported more than one comparison, resulting in an overlap between some of the controls included in studies on AN and those included in studies with BN [50, 58, 86]. To calculate the raw, untransformed mean performance of the controls, we excluded three studies: because they used the children version of the RMET [87, 95]; or because the authors had recalculated the global score by excluding some items from the RMET [91]. However, these studies were included in the subsequent pairwise meta-analysis since it relates to standardized mean differences, which are less likely to be affected by the version of the tool.

Mean scores on controls on the RMET were close to 27 in both the controls of AN studies (Figure A1 in supplementary material) and those of BN studies (Figure A2 in supplementary material).

**Fig. 2:**
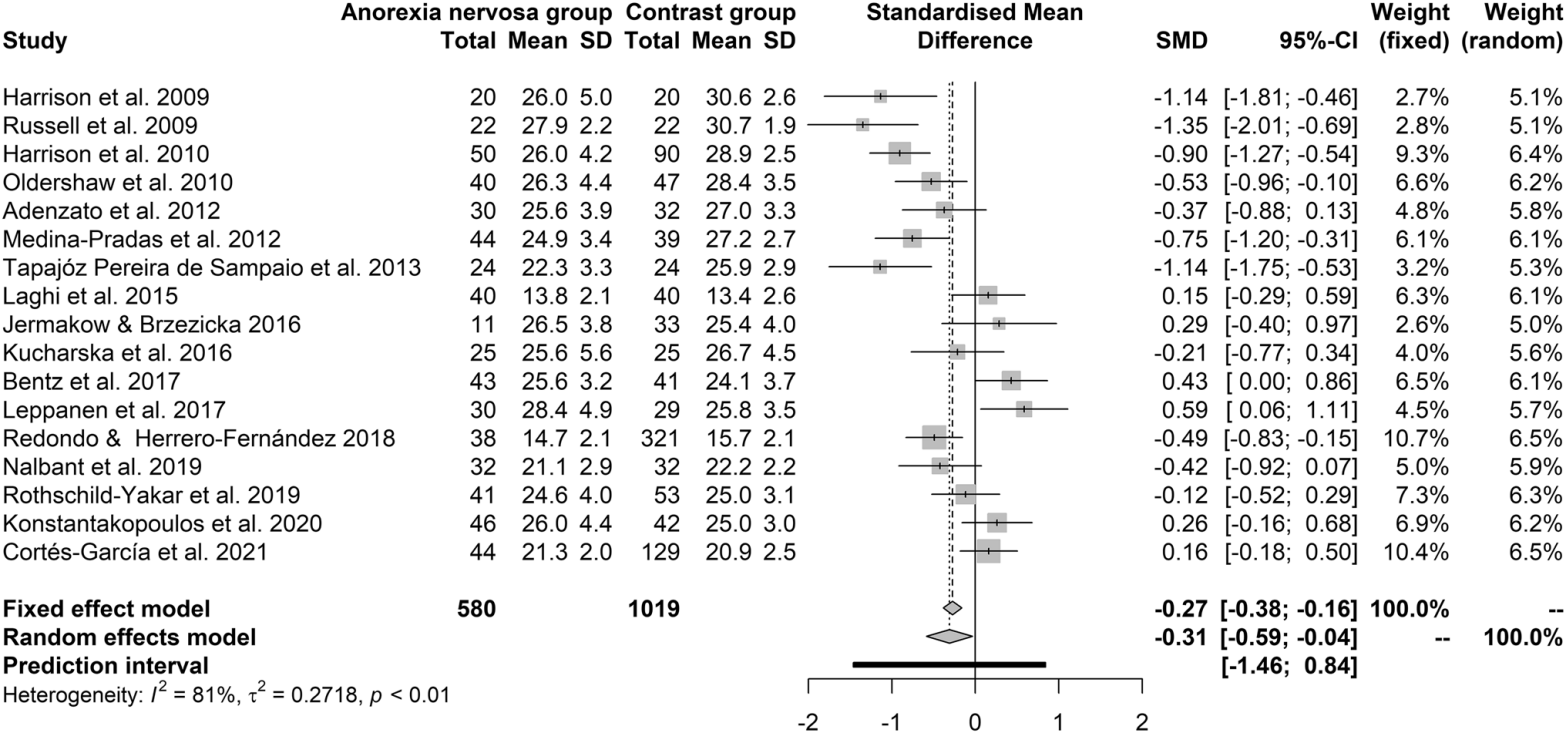
Forest plot of the effect sizes of the RMET scores' differences, calculated as Hedges’ g, in the comparison between patients with active AN and controls

Among cases, mean scores on the RMET ranged 21 to 28 (mean = 25) in patients with active AN; 24 to 28 (mean = 25) in patients with recovered AN; 24 to 27 (mean = 25) in patients with BN; 24 to 27 (mean = 25) in patients with EDNOS; and they were found to be 20 (range: 19.9 to 20.3) in the two studies including patients with BED and obesity.

### Performances on the RMET of patients with AN

Overall, 17 samples were included, summarizing the results from 580 patients with active AN and 1019 controls. Ten studies found no differences between patients with active AN and controls, while seven studies found impaired performance on the RMET of patients with active AN. Overall, patients with AN in the active phase showed worse performances on the RMET than gender- and age-matched controls (details in Table 2).

**Table 2:** Main results of the meta-analysis of studies on performances on the RMET of patients with eating disorders

		*k*	*n*	Effect size	95% CI		*z*	*p*	*Q*	*p* value	tau^2^ (%)	*I*^*2*^ (%)	95% CI	
Active anorexia nervosa	FE model	17	1599	− 0.27	− 0.38	− 0.16	− 4.85	0.0001						
	RE model	17	1599	− 0.31	− 0.58	− 0.04	− 2.22	0.0267	83.45	< 0.001	0.272	81	70	88
	RE model after trim-and-fill	17	1643	− 0.25	− 0.54	0.03	− 1.74	0.0827	94.51	< 0.001	0.322	82	72	88
	FE model without outliers	15	1415	− 0.17	− 0.29	− 0.05	− 2.86	0.0042						
	RE model without outliers	15	1415	− 0.20	− 0.47	0.06	− 1.52	0.1280	58.78	< 0.001	0.212	76	61	86
Recovered anorexia nervosa	FE model	3	268	− 0.34	− 0.60	− 0.09	− 2.63	0.0086						
	RE model	3	268	− 0.31	− 0.77	0.15	− 1.30	0.1926	6.76	0.0340	0.114	70	0	91
Bulimia nervosa	FE model	5	434	− 0.38	− 0.58	− 0.19	− 3.87	0.0001						
	RE model	5	434	− 0.35	− 072	0.01	− 1.90	0.0572	12.90	0.0118	0.121	69	20	88
Binge eating disorder	FE model	2	106	− 0.12	− 0.26	0.51	− 0.64	0.52						
	RE model	2	106	− 0.12	− 0.26	0.51	− 0.64	0.52	0.27	0.6066	0.0	0	0	0
EDNOS	FE model	2	153	− 0.55	− 0.89	− 0.22	− 3.23	0.0013	9.1					
	RE model	2	153	− 0.61	− 1.26	0.04	− 1.84	0.0664	3.56	0.0592	0.159	72	0	94

*k* number of studies, *n* overall number of participants, *FE* fixed-effects model, *RE* random-effects model

For each diagnosis, an independent meta-analysis has been carried out. For each diagnosis, both the fixed-effects and the random-effects estimates have been reported, with sensitivity analysis when there were enough studies

The random-effects model was more conservative than the fixed-effects model, i.e., it had a larger 95% CI, with the lower bound approaching zero; the prediction interval indicated that the finding would not hold in future studies with the same trend as those included in the analysis (Fig. 2).

**Fig. 3:**
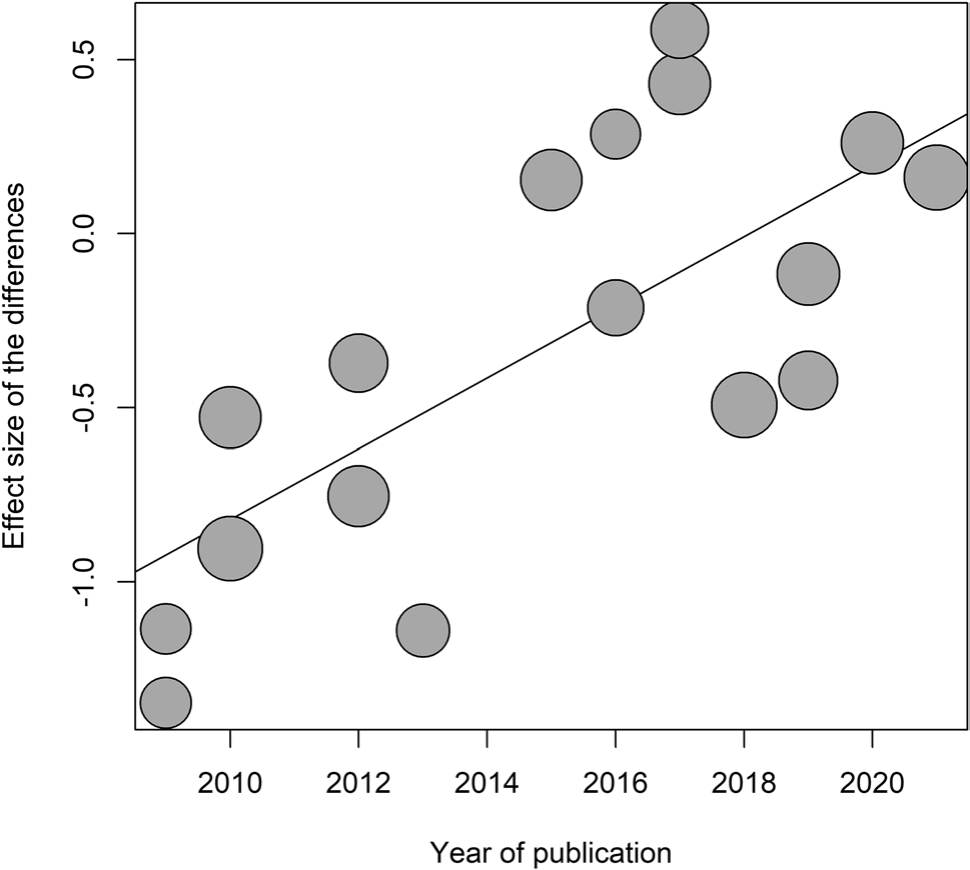
Bubble plot of the result of the meta-regression of the year of publication (horizontal axis) on the effect sizes of the RMET

Heterogeneity was substantial in this meta-analysis, likely as a reflection of differences among the samples in terms of duration of illness, type of the disorder (restrictive variant versus binge-purge variant), and stage of the condition (first-episode versus recurrence or chronicity of the episode). Earlier studies were more likely to find worse performances on the RMET of patients with active AN than later studies. Indeed, the year of publication accounted for 61% of the heterogeneity in the meta-analysis and each year imported a displacement towards better performances by 0.10 (95% CI: 0.05 to 0.15) in the differences between patients with active AN and controls (Fig. 3).

Age (Beta = − 0.05; s.e. Beta = 0.03; *z* = − 1.84; *p* = 0.066), BMI (Beta = − 0.07; s.e. Beta = 0.08; *z* = 0.40; *p* = 0.688), and quality of the studies (*Q* = 2.88, *p* = 0.236) did not impact on estimates of the differences on the RMET between patients with active AN and controls. Conversely, criteria for diagnosis (Q = 9.66, p = 0.022) and duration of illness (Beta = − 0.09; s.e. Beta = 0.04; *z* = − 2.49; *p* = 0.0127) were related to the estimates and explained, respectively, 37% and 43% of heterogeneity. This was in part a consequence of their relationship with the year of publication, with longer duration of illness in samples of earlier studies (correlation between duration of illness and year of publication: *r* = − 0.58, *p* = 0.048), and studies based on DSM-IV (seven studies) more likely to find differences and larger effect size (random-effects model estimates: − 0.70 [− 1.06 to − 0.34]) than studies based on DSM-IV-TR (five studies; estimates: − 0.21 [− 0.62 to 0.20]), DSM-5 (three studies; estimates: 0.006 [− 0.53 to 0.55]), or ICD-10 (two studies; estimates: 0.37 [− 0.32 to 1.06]).

Egger’s regression test did not reveal publication bias (*t* = − 0.759; df = 15; *p* = 0.46), however, the trim-and-fill method suggested one study to be filled to gain symmetry at the funnel plot on the opposite side of the studies of Russell et al. [82] (Figure A3 in supplementary material). After reanalysis of the data with the imputed study, the random-effects model was no longer statistically significant: the estimates from 18 studies were − 0.25 (95% CI: − 0.54 to 0.03); *z* = − 1.74; *p* = 0.083. Heterogeneity remained substantial: *I*^*2*^ = 82% (95% CI: 72–88%).

The radial plot indicated that several studies scored outside two standard deviations from the mean in the sample, with the studies of Russell et al. [82] and Harrison et al. [83] being four standard deviations below the mean in the sample (Figure A4 in supplementary material). When the analysis was repeated by excluding these two outliers, the mean difference dropped to 0.17 in the fixed-effects model and became statistically not significant in the random-effects model (see Table 2 and Figure A5 in supplementary material).

**Fig. 4:**
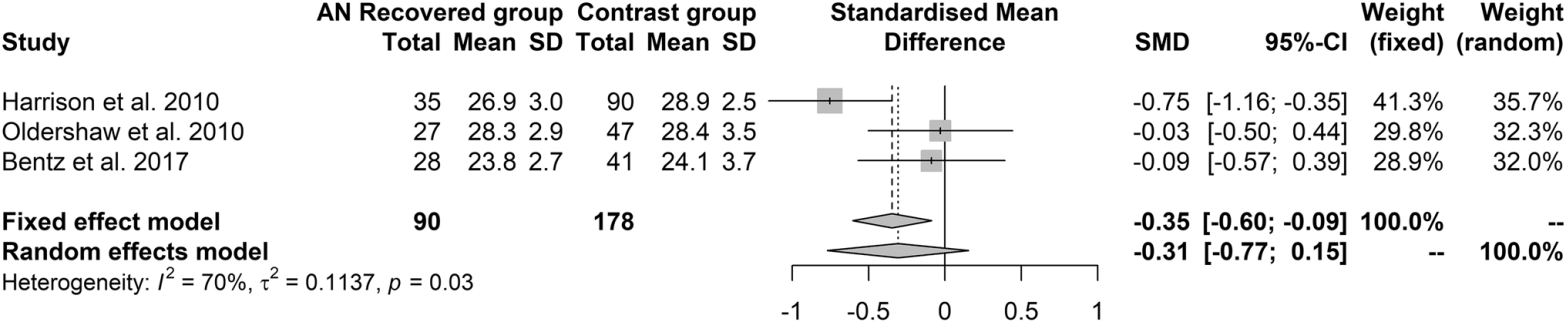
Forest plot of the effect sizes of the RMET scores' differences, calculated as Hedges’ g, in the comparison between patients with recovered AN and controls

The analysis of the three samples of patients with recovered AN confirmed a poorer performance in the patients than in controls at the fixed-effects but not at the random-effects model (Table 2 and Fig. 4).

### Performances on the RMET of patients with BN

**Fig. 5:**
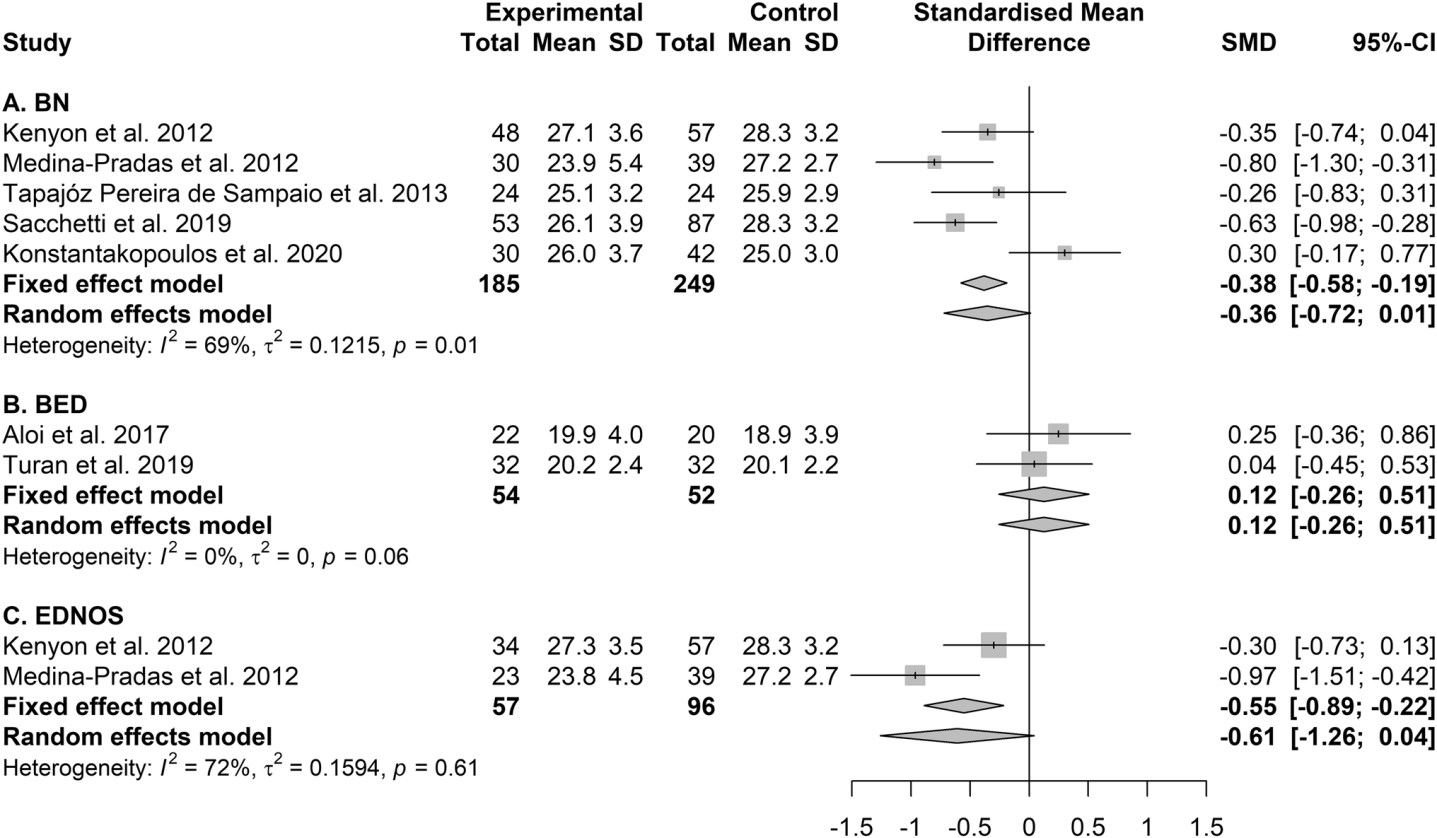
Forest plot of the effect sizes of the RMET scores' differences, calculated as Hedges’ g, in the comparison between patients with BN (section **A**), BED (section **B**),

As for the patients diagnosed with BN, 5 samples were included, summarizing results from 185 patients with active BN and 249 controls. Two studies reported poorer performances of patients with BN than controls; three studies found no statistically significant differences. The meta-analysis suggested poorer performances of patients with BN than controls at the fixed-effects model, with similar effect size than in studies on patients with AN, but the random-effects model indicated that the findings did not hold when heterogeneity—which was moderate to substantial—was taken into account (Table 2 and Fig. 5, section A).

### Performances on the RMET of patients with BED

Two studies explored the performances of patients with BED on the RMET and found no statistically significant differences between 54 cases with obesity versus 52 controls with obesity but without BED (Table 2 and Fig. 5, section B).

### Performances on the RMET of patients with EDNOS

Two studies included patients with EDNOS and summarized results from 57 cases, and 96 controls. One study showed poorer performances of patients than controls and the other reported no statistically significant differences. The meta-analysis of the studies including patients with EDNOS reached the statistically significant threshold on the fixed-effects model but not on the random-effects model, with moderate to substantial heterogeneity (Table 2 and Fig. 5, section C).

## Discussion

At first sight, this meta-analysis found that patients with eating disorders perform worse than putatively healthy controls on a task aimed at measuring the understanding of others’ mental states. However, the estimated differences had small effect sizes, which ranged from medium to negligible in the confidence interval. The differences from controls on the RMET were more clear-cut in studies including patients with active AN than in those including patients with recovered AN, or including patients with BN, and were minimal or absent in studies including patients with BED or EDNOS. The greater is the chance of malnutrition and its impact on brain functions [96, 97], the higher the difference between patients with eating disorders and controls. Unfortunately, studies with direct comparison by diagnosis were too few to allow meaningful analyses. Moreover, several caveats have to be taken into account to reach a full grasp of these results.

When the heterogeneity of the samples is taken into account (i.e., on the basis of the random-effects model), patients with BN, EDNOS and BED do not differ from controls. As for the patients with AN, the exclusion of the most extreme samples (the so-called outliers) makes the results of the random-effects model no longer significant. Moreover, the so-called prediction interval suggests that the findings of the meta-analysis concerning the patients with AN will not hold in future studies with the same characteristics as those included in the meta-analysis. Thus, patients with AN can show worse performances on the RMET than controls, but this depends more on some characteristics of the disorder (restrictive variant versus binge-purge variant; first-episode versus recurrent) than on their being a core symptom of the disorder itself. Studies did not offer enough details to perform subgroup analysis at the level that would be required to identify subgroups of patients with AN expressing insufficient performances on the RMET. For example, some studies found a relation between alexithymia and RMET performances, with worse performances in subjects with higher scores on a measure of alexithymia [17, 98]. Alexithymia was also suggested to affect emotion recognition [99], and emotion recognition contributes to the performances on the RMET. However, we were unable to retrieve enough studies with information on alexithymia in the investigated samples to undertake a meta-regression and evaluate the role of alexithymia in the patients’ performances on the RMET, or its impact on the heterogeneity of the findings.

Overall, the analyses were all characterized by moderate to substantial heterogeneity (according to the calculated 95% CI of the I^2^), but age, BMI, and quality of the studies did not impact estimates nor decreased the heterogeneity. The most remarkable impact on heterogeneity was from the year of the publication, which might suggest that earlier studies were published more often when they reported a difference between cases and controls, while the reverse is probably true for the most recent studies. Indeed, while Egger’s test did not reveal publication bias, the trim-and-fill method identified some asymmetry in the funnel plot. It should be taken into account that changes have occurred in the criteria for diagnosing eating disorders, and samples including patients diagnosed with AN or BN according to the DSM-5 may include less severe cases than those included in samples that were diagnosed according to the more restrictive criteria of the DSM-IV. Indeed, there is some evidence that cases that would have been classified under the label of EDNOS according to the DSM-IV are now diagnosed under one major category (AN, BN, BED) according to the DSM-5 [59]. This could be a reason why the studies using DSM-IV criteria were more likely to report that patients with AN perform worse than controls on the RMET.

Overall, this meta-analysis indicates that patients with eating disorders do not perform poorer on the RMET than gender- and age-matched healthy controls. However, patients with active AN may show some impairment on the RMET, thus revealing an impact of the disorder on the capacity of understanding others’ mental state, which could affect the social functioning of the patients and might negatively influence their paths to recovery [43, 100]. There could be reasons for patients with active AN to perform poorly on a tool aimed at measuring other people’s mental states. In patients with active AN, several studies based on diffusion tensor imaging reported white matter microstructural abnormalities of the left superior longitudinal and inferior frontal-occipital fasciculi, some of the bundles that connect the frontal lobe with temporal and occipital lobes [101]. The same structures were also activated during the RMET task [102]. Another region implicated in the RMET task, the inferior frontal gyrus [103], was reported to show alterations in patients with acute AN [104]. Finally, the default mode network has been consistently related to the social understanding of others’ mental states [105], and has been reported to be altered in patients with acute AN [106]. Moreover, functional neuroimaging studies in patients with acute AN have shown abnormalities in the frontal visual system, the attention network, and the arousal and emotional processing systems [107], all systems contributing to the execution of the RMET task.

Some of the abnormalities reported in acute AN tend to reverse after remission of symptoms, in particular alterations in white matter microstructures [108]. Studies on patients with BN and BED reported primarily alterations in circuits involved in appetite, the processing of food stimuli, and impulse control rather than in circuits involved in the understanding of others’ mental states [109], while structural and functional abnormalities are generally less extensive and pervasive in patients with BN or BED than in those with AN. Overall, brain imaging studies in patients with eating disorders are hampered by small sample sizes, inconsistencies in methods, and large variability in samples’ characteristics [107–109]. Small sample sizes and large variability in samples’ characteristics also affect studies on affective cognition in eating disorders [46, 62]. However, it is suggestive that abnormalities in circuits that are involved in the understanding of others’ mental states are more often reported in patients with AN than in patients with BN or BED. This would be consistent with greater impairment in the RMET task in patients with AN than in patients with BN, BED or EDNOS. The likely state-dependence (because of malnutrition) of some of these abnormalities would justify better performances of patients with recovered AN than in those with acute or chronic AN.

## Strength and limits

The major strengths of the study are the use of state-of-the-art methods to perform the meta-analysis and the retrieval of a larger sample of studies and participants than in the past meta-analyses on the topic. Several limitations should be also considered. Most studies had sample sizes lower than 50, and this impacted on the statistical power to find differences and apply sensitivity analyses. Participants were women in their majority, thus nothing can be said about men with eating disorders, who represent a non-negligible fraction of the cases with eating disorders, with lifetime prevalence estimated at 0.74% (95% CI: 0.24–1.52) [110]. Some relevant information—such as BMI, age of onset, or severity—was not available for all studies. Moreover, few studies were available for some specific diagnoses, thus potentially limiting the generalizability of the results. Finally, heterogeneity was high in most comparisons, and it was partially explained by year of publication, criteria for diagnosis, and duration of illness, which were interrelated to each other, limiting a clear understanding of the contribution of each element.

## What is already known on this subject?

A deficit in the understanding of others' mental states, the so-called ToM, has been reported in patients with AN. Less information is available concerning other eating disorders. ToM deficits can have a role in social impairment, poor insight, and resistance to treatment that are observed in patients with eating disorders.

## What does this study add?

This meta-analysis shows that patients with eating disorders do not suffer from an impaired understanding of others’ mental states, with the exception of a still-to-be-identified subgroup of patients with active AN. Indeed, patients diagnosed with an active illness and who are more physically impaired because of malnutrition would exhibit poorer performance on the RMET. Publication bias might have emphasized the presence of poorer performances in patients with AN on the RMET, a recognized measure of ToM. Moreover, studies have several limitations in terms of both sample size—hence of statistical power—and data description.

## Conclusions

Taking into account the results of the meta-analysis and the limitations of the included studies, it can be concluded that patients with eating disorders do not suffer from an impaired understanding of others’ mental states, with the exception of a still-to-be-identified subgroup of patients with acute AN. Three main gaps should be filled in the investigation of affective cognition in eating disorders: (1) the quality of the studies should be improved, in terms of both the method and sample size; (2) more data are necessary on patients with BN, BED, or other specified feeding or eating disorder (OSFED); (3) there is a need for direct comparison between diagnoses, to confirm that the performances on the RMET are principally impaired in patients with acute AN when compared to patients with recovered AN, or with BN, BED or OSFED. Larger sample sizes and the direct comparison among diagnoses would also allow the evaluation of the role of comorbidity, especially for social anxiety and depression [111], which might affect the performances on the RMET [112, 113].

## Supplementary Information

Below is the link to the electronic supplementary material.

Supplementary file1 (DOCX 937 KB)

## Data Availability

The corresponding author had full access to all the data in the study and takes responsibility for the integrity of the data and the accuracy of data analysis. Data sharing is not applicable to this article as no new data were created or analyzed in this study. Data that were extracted from the included studies are depicted in the tables and the figures of this article.

## Code availability

Standard codes were used in the meta-analysis. Sample codes will be shared upon request.
