# Supplementary material for: Affective cognition in eating disorders: a systematic review and meta-analysis of the performance on the “Reading the Mind in the Eyes” Test
Source: Eat Weight Disord. 2022 Apr 6;27(7):2291–307. doi: 10.1007/s40519-022-01393-8 (PMC9556412; doi:10.1007/s40519-022-01393-8)
Supplement: Supplementary file 1 — Supplementary file1 (DOCX 937 KB) [file 40519_2022_1393_MOESM1_ESM.docx]

List of excluded articles

1. Calvo Sagardoy R, Gallego Morales LT, Solórzano Ostolaza G, Kassem García S, Morales Martínez C, Codesal Julián R, Blanco Fernández A. Procesamiento emocional en pacientes con trastornos de conducta alimentaria adultas vs adolescentes; reconocimiento emocional y teoria de la mente [Emotional processing in adult vs adolescent patients with eating behavior disorders; emotional recognizing and the mental theory]. Nutr Hosp. 2014 Apr 1;29(4):941-52. Spanish. doi: 10.3305/nh.2014.29.4.7326.
2. Harrison A, Sullivan S, Tchanturia K, Treasure J. Emotional functioning in eating disorders: attentional bias, emotion recognition and emotion regulation. Psychol Med. 2010 Nov;40(11):1887-97. doi: 10.1017/S0033291710000036.
3. Kanakam N, Krug I, Raoult C, Collier D, Treasure J. Social and emotional processing as a behavioural endophenotype in eating disorders: a pilot investigation in twins. Eur Eat Disord Rev. 2013 Jul;21(4):294-307. doi: 10.1002/erv.2232.
4. Naor-Ziv R, Glicksohn J. Investigating Cognitive Deficits as Risk Factors for Developing Eating Disorders During Adolescence. Dev Neuropsychol. 2016 Jan-Mar;41(1-2):107-24. doi: 10.1080/87565641.2016.1170129.
5. Oldershaw A, Treasure J, Hambrook D, Tchanturia K, Schmidt U. Is anorexia nervosa a version of autism spectrum disorders? Eur Eat Disord Rev. 2011 Nov-Dec;19(6):462-74. doi: 10.1002/erv.1069.
6. Rothschild-Yakar L, Goshen D, Enoch-Levy A, Raanan H, Gur E, Stein D, Golan O. General mentalizing, emotional theory of mind and interpersonal mistrust in anorexia nervosa: The validation of the Hebrew version of the Cambridge mindreading face-task. Clin Psychol Psychother. 2021 Jun 10. doi: 10.1002/cpp.2626.
7. Tapajóz F, Soneira S, Catoira N, Aulicino A, Allegri RF. Impaired theory of mind in unaffected first-degree relatives of patients with anorexia nervosa. Eur Eat Disord Rev. 2019 Nov;27(6):692-699. doi: 10.1002/erv.2701.
8. Tapajóz Pereira de Sampaio F, Soneira S, Aulicino A, Allegri RF. Theory of mind in eating disorders and their relationship to clinical profile. Eur Eat Disord Rev. 2013 Nov;21(6):479-87. doi: 10.1002/erv.2247.
9. Tchanturia K, Happé F, Godley J, Treasure J, Bara-Carril N, Schmidt U. ‘Theory of mind’ in anorexia nervosa. Eur. Eat. Disorders Rev. 2004; 12: 361-366. https://doi.org/10.1002/erv.608
10. Zegarra-Valdivia JA, Chino-Vilca BN. Social Cognition and Executive Function Impairment in Young Women with Anorexia Nervosa. Clínica y Salud 2018; 29(3): 107-113. https://dx.doi.org/10.5093/clysa2018a16
11. Boscoe A, Stanbury R, Harrison A. Social-emotional functioning in young people with symptoms of eating disorders: A gender inclusive analogue study. Brain Behav. 2021 Mar;11(3):e02017. doi: 10.1002/brb3.2017.
12. Bremser JA, Gallup GG Jr. From one extreme to the other: negative evaluation anxiety and disordered eating as candidates for the extreme female brain. Evol Psychol. 2012 Aug 2;10(3):457-86. https://doi.org/10.1177/147470491201000306

Key to Figures captions

Fig. 1. PRISMA Flow chart

Fig. 2. Forest plot of the effect sizes of the RMET scores' differences, calculated as Hedges’ g, in the comparison between patients with active AN and controls.

Fig. 3. Bubble plot of the result of the meta-regression of the year of publication (horizontal axis) on the effect sizes of the RMET scores' differences (vertical axis).

Fig. 4. Forest plot of the effect sizes of the RMET scores' differences, calculated as Hedges’ g, in the comparison between patients with recovered AN and controls.

Fig. 5. Forest plot of the effect sizes of the RMET scores' differences, calculated as Hedges’ g, in the comparison between patients with BN (section A), BED (section B), and EDNOS (section C) and controls.

Fig. A1. Forest plot of the raw, untransformed mean of the RMET scores in samples of controls of studies concerning patients with AN.

Fig. A2. Forest plot of the raw, untransformed mean of the RMET scores in samples of controls of studies concerning patients with BN.

Fig. A3. Funnel plot of the effect sizes of the RMET scores' differences in the comparison between patients with active AN and controls. The mean standardized difference, calculated as Hedges’ g, is reported in the horizontal axis, while standard error is reported in the vertical axis. The funnel plot also reports the additional study that the trim-and-fill method suggested to be added to make the plot symmetrical.

Fig. A4. Radial plot of the standardized effect (in z-scores, on the vertical axis) against the inverse of standard error (on the horizontal axis) in studies comparing patients with active AN and controls.

Fig. A5. Forest plot of the effect sizes of the RMET scores' differences, calculated as Hedges’ g, in the comparison between patients with active AN and controls after the exclusion of two outliers studies.

Table A. Quality assessment of the included studies

| Study | 1 | 2 | 3 | 4 | 5 | 6 | 7 | 8 | 9 | 10 | 11 | 12 | Rating |
| --- | --- | --- | --- | --- | --- | --- | --- | --- | --- | --- | --- | --- | --- |
| Harrison et al. 2009 | Y | Y | N | CD | CD | Y | N | Y | CD | Y | N | CD | Poor |
| Russell et al. 2009 | Y | Y | N | CD | CD | Y | N | Y | CD | Y | N | Y | Fair |
| Harrison et al. 2010 | Y | Y | N | CD | CD | Y | N | Y | CD | Y | N | Y | Fair |
| Oldershaw et al. 2010 | Y | Y | N | CD | CD | Y | N | Y | CD | Y | N | Y | Fair |
| Adenzato et al. 2012 | Y | Y | N | CD | CD | Y | N | Y | CD | Y | N | N | Poor |
| Kenyon et al. 2012 | Y | Y | N | CD | CD | Y | N | Y | CD | Y | N | Y | Fair |
| Medina-Pradas et al. 2012 | Y | Y | N | CD | CD | Y | N | Y | CD | Y | N | Y | Fair |
| Tapajóz Pereira de Sampaio et al. 2013 | Y | Y | N | CD | CD | Y | N | Y | CD | Y | N | N | Poor |
| Laghi et al. 2015 | Y | Y | N | CD | CD | Y | N | Y | CD | Y | N | N | Poor |
| Jermakow & Brzezicka 2016 | Y | Y | N | CD | CD | Y | N | Y | CD | Y | N | CD | Poor |
| Kucharska et al. 2016 | Y | Y | Y | CD | CD | Y | N | Y | CD | Y | N | Y | Good |
| Aloi et al. 2017 | Y | Y | N | Y | Y | Y | N | Y | CD | Y | N | N | Good |
| Bentz et al. 2017 | Y | Y | N | CD | CD | Y | N | Y | CD | Y | N | N | Poor |
| Leppanen et al. 2017 | Y | Y | Y | CD | CD | Y | Y | Y | CD | Y | Y | Y | Good |
| Redondo & Herrero-Fernández 2018 | Y | Y | N | CD | CD | Y | N | Y | CD | Y | N | Y | Fair |
| Nalbant et al. 2019 | Y | Y | N | Y | Y | Y | N | Y | CD | Y | N | Y | Good |
| Rothschild-Yakar et al. 2019 | Y | Y | Y | CD | Y | Y | N | Y | CD | Y | Y | Y | Good |
| Sacchetti et al. 2019 | Y | Y | N | CD | CD | Y | N | Y | CD | Y | N | CD | Poor |
| Turan et al. 2019 | Y | Y | Y | Y | Y | Y | N | Y | CD | Y | N | CD | Good |
| Konstantakopoulos et al. 2020 | Y | Y | N | CD | CD | Y | N | Y | CD | Y | N | Y | Fair |
| Cortés-García et al. 2021 | Y | Y | N | CD | CD | Y | N | Y | CD | Y | N | CD | Poor |

Y=Yes; N=No; CD=cannot determine; NA=not applicable; NR=not reported

Questions

1. Was the research question or objective in this paper clearly stated and appropriate?

2. Was the study population clearly specified and defined?

3. Did the authors include a sample size justification?

4. Were controls selected or recruited from the same or similar population that gave rise to the cases (including the same timeframe)?

5. Were the definitions, inclusion and exclusion criteria, algorithms or processes used to identify or select cases and controls valid, reliable, and implemented consistently across all study participants?

6. Were the cases clearly defined and differentiated from controls?

7. If less than 100 percent of eligible cases and/or controls were selected for the study, were the cases and/or controls randomly selected from those eligible?

8. Was there use of concurrent controls?

9. Were the investigators able to confirm that the exposure/risk occurred prior to the development of the condition or event that defined a participant as a case?

10. Were the measures of exposure/risk clearly defined, valid, reliable, and implemented consistently (including the same time period) across all study participants?

11. Were the assessors of exposure/risk blinded to the case or control status of participants?

12. Were key potential confounding variables measured and adjusted statistically in the analyses? If matching was used, did the investigators account for matching during study analysis?


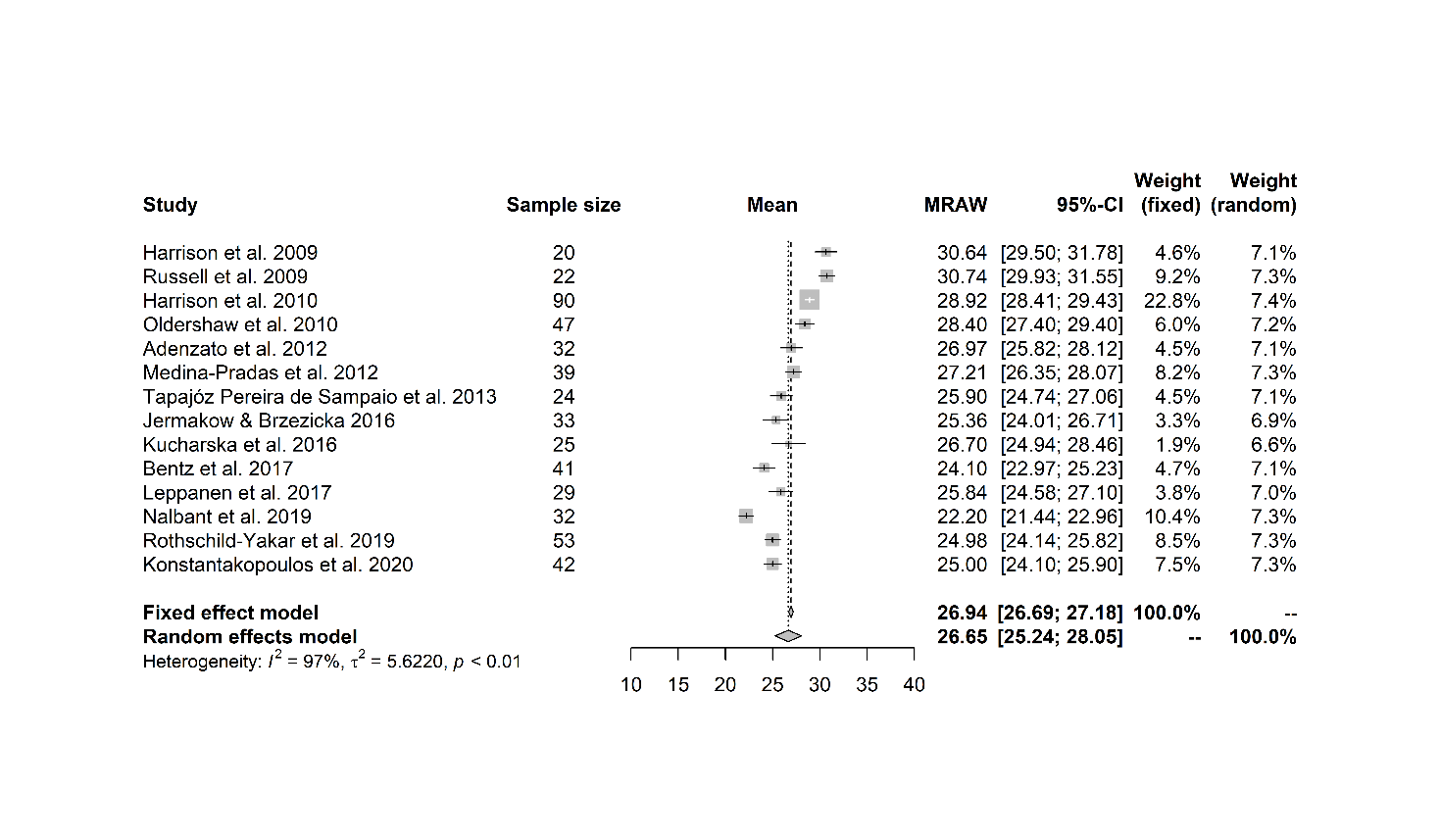


Fig. A1. Forest plot of the raw, untransformed mean of the RMET scores in samples of controls of studies concerning patients with AN.


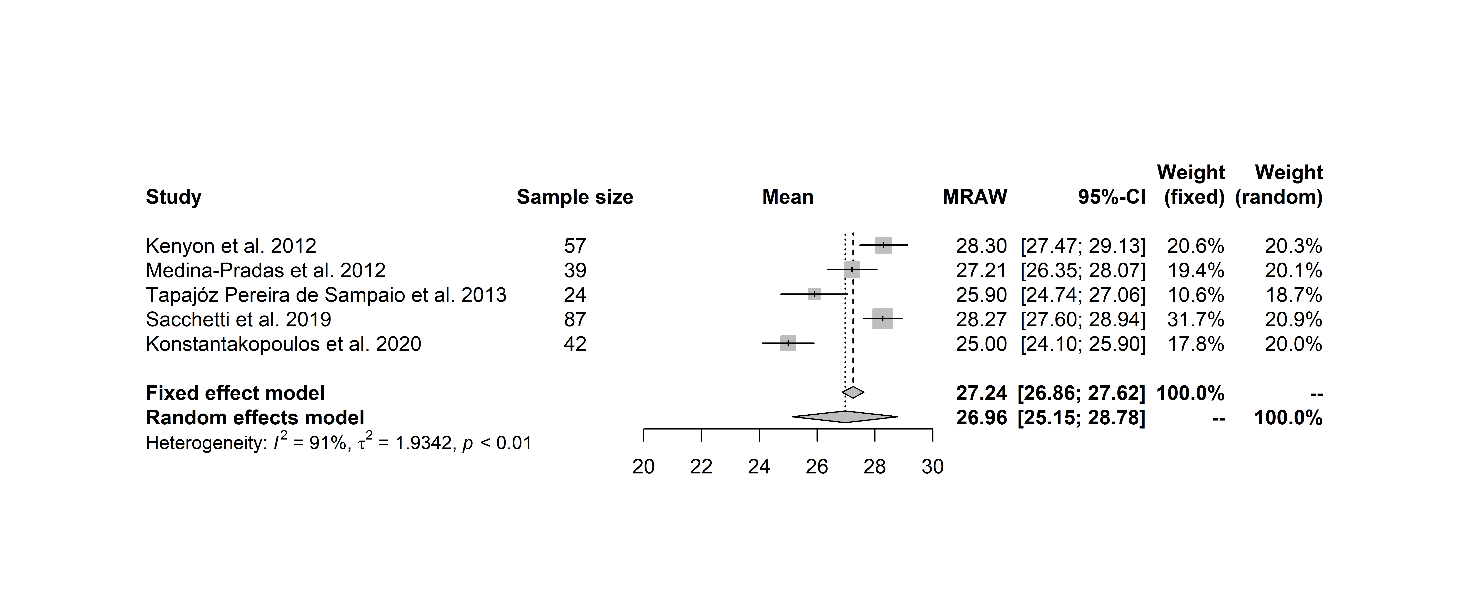


Fig. A2. Forest plot of the raw, untransformed mean of the RMET scores in samples of controls of studies concerning patients with BN.


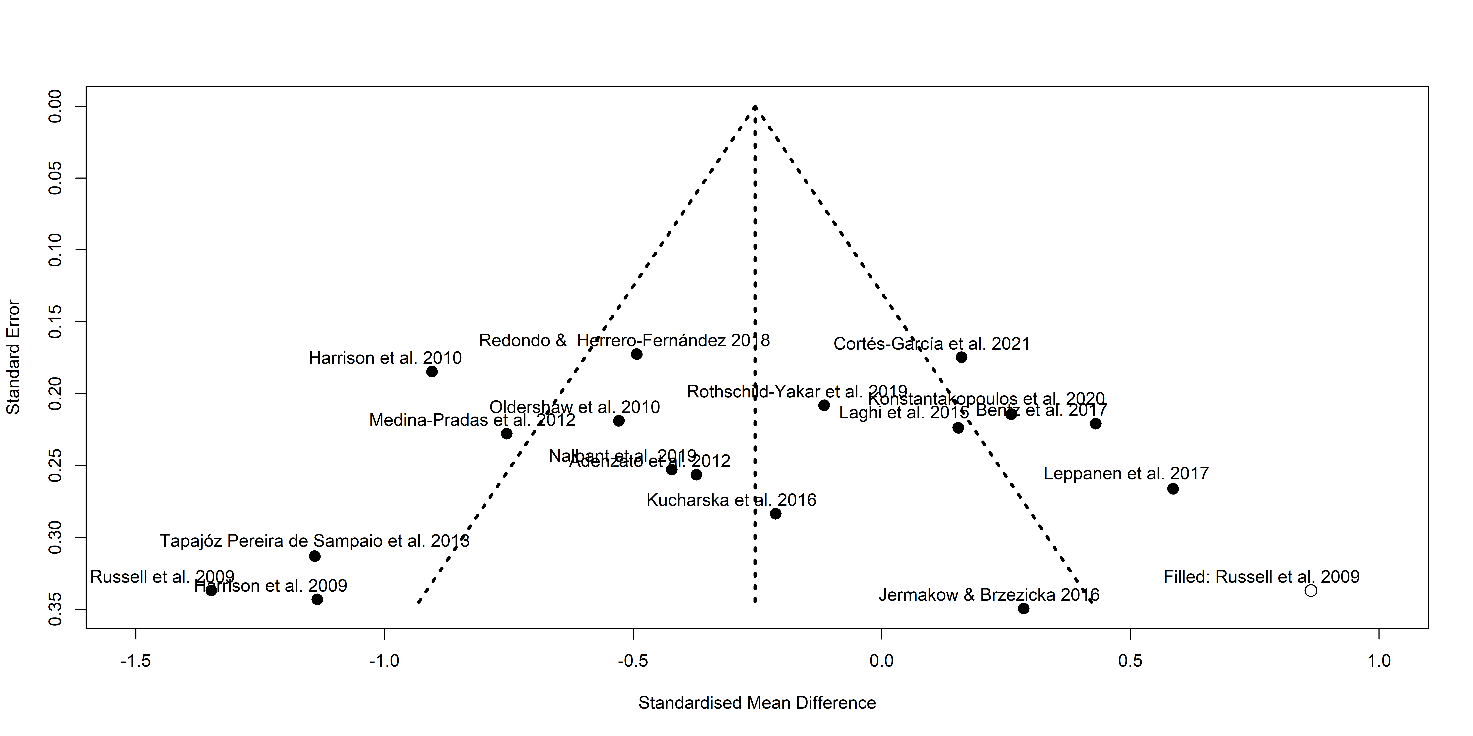


Fig. A3. Funnel plot of the effect sizes of the RMET scores' differences in the comparison between patients with active AN and controls. The mean standardized difference, calculated as Hedges’ g, is reported in the horizontal axis, while standard error is reported in the vertical axis. The funnel plot also reports the additional study that the trim-and-fill method suggested to be added to make the plot symmetrical.


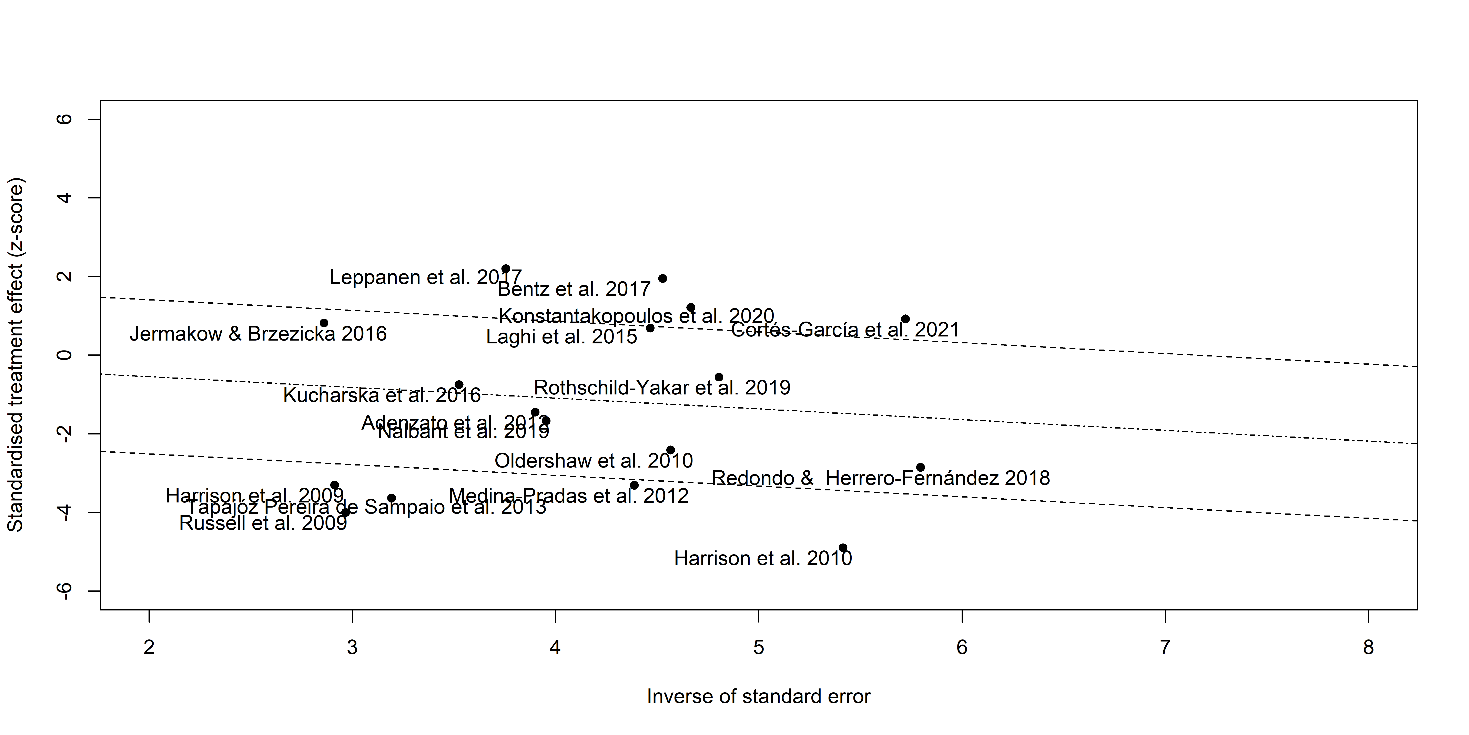


Fig. A4. Radial plot of the standardized effect (in z-scores, on the vertical axis) against the inverse of standard error (on the horizontal axis) in studies comparing patients with active AN and controls.


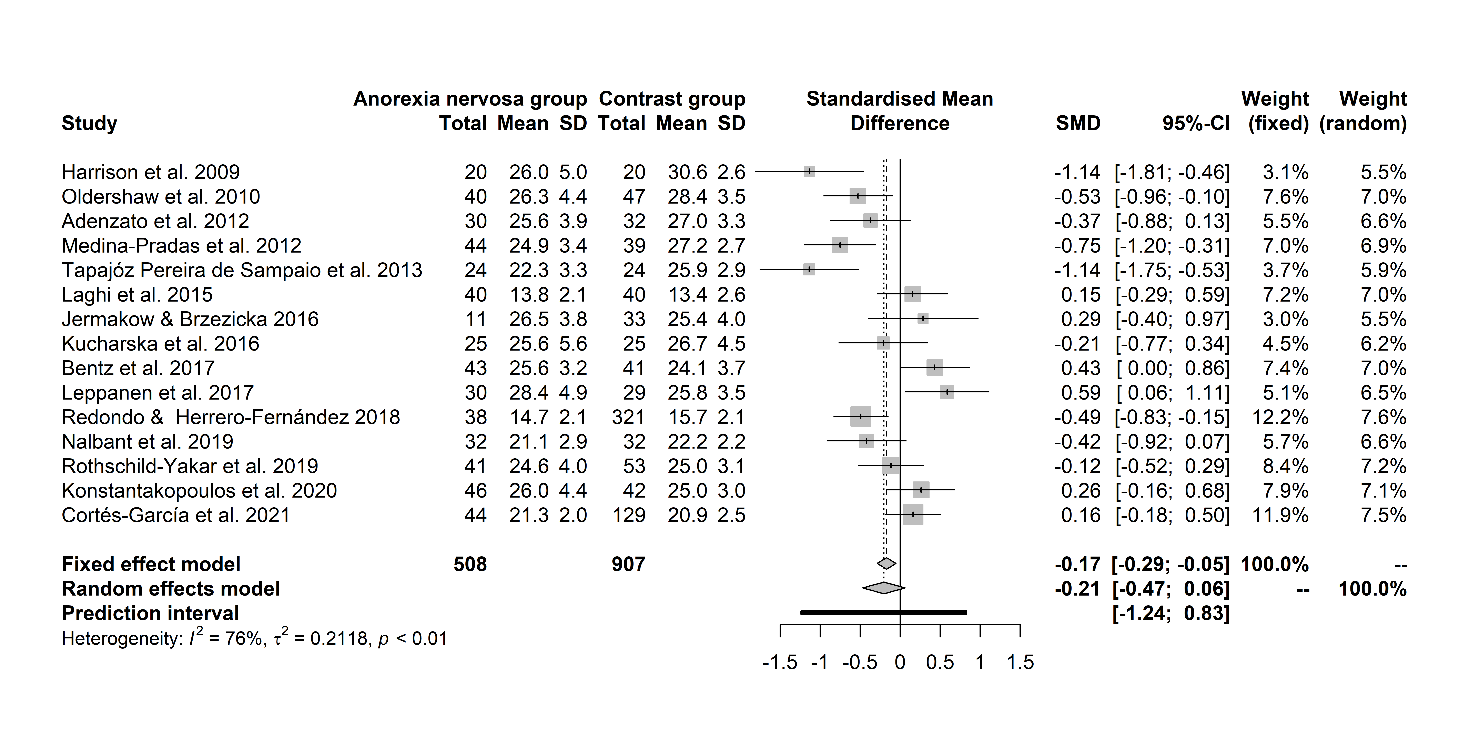


Fig. A5. Forest plot of the effect sizes of the RMET scores' differences, calculated as Hedges’ g, in the comparison between patients with active AN and controls after the exclusion of two outliers studies.
